# Erythropoietin and Its Angiogenic Activity

**DOI:** 10.3390/ijms18071519

**Published:** 2017-07-13

**Authors:** Patrícia Kimáková, Peter Solár, Zuzana Solárová, Radovan Komel, Nataša Debeljak

**Affiliations:** 1 Laboratory of Cell Biology, Institute of Biology and Ecology, Faculty of Science, Pavol Jozef Šafárik University in Košice, Košice 04001, Slovak; kimakova.patricia@gmail.com; 2 Institute of Pharmacology, Faculty of Medicine, P.J. Šafárik University in Košice, Košice 04001, Slovak; zuzana.solarova@upjs.sk; 3 Medical Centre for Molecular Biology, Institute of Biochemistry, Faculty of Medicine, University of Ljubljana, Ljubljana SI-1000, Slovenia; komel@mf.uni-lj.si (R.K.); natasa.debeljak@mf.uni-lj.si (N.D.)

**Keywords:** erythropoietin, erythropoietin receptor, endothelial, angiogenesis, cancer

## Abstract

Erythropoietin (EPO) is the main hematopoietic hormone acting on progenitor red blood cells via stimulation of cell growth, differentiation, and anti-apoptosis. However, its receptor (EPOR) is also expressed in various non-hematopoietic tissues, including endothelium. EPO is a pleiotropic growth factor that exhibits growth stimulation and cell/tissue protection on numerous cells and tissues. In this article we review the angiogenesis potential of EPO on endothelial cells in heart, brain, and leg ischemia, as well as its role in retinopathy protection and tumor promotion. Furthermore, the effect of EPO on bone marrow and adipose tissue is also discussed.

## 1. Introduction

Erythropoietin (EPO) is the main hematopoietic cytokine that regulates the formation of red blood cells in the process of hematopoiesis [1]. The main source of EPO after fetal development is the liver [2], while in adults the major source is the kidneys [3]. The effect of EPO is mediated by its interaction with the EPO receptor (EPOR), which is a member of the cytokine receptor family. EPOR is preferentially expressed in erythroid cells, but also in many non-hematopoietic cells, including vascular endothelial cells (ECs) and cancer cells [4]. Formation of the complex EPO/EPOR results in the activation of proteins involved in basic signal transduction [5] such as Janus kinase 2 (JAK-2) and signal transducer and activator of transcription (STAT) [6], and of other signal pathways that control proliferation, survival of cells, and gene expression [7]. In this regard, EPO robustly induces phosphorylation of STAT-5 in human umbilical vein endothelial cells (HUVECs), but only very weakly in smooth muscle cells, indicating the difference between cells [8]. Interestingly, the signaling of EPO in ECs is mediated via phosphorylation of STAT-5 similar to that occurring in erythroid cells [9]. Indeed, EPO via EPOR enhances proliferation and migration of HUVECs and bovine adrenal capillary ECs, as was demonstrated by the presence of EPOR mRNA in HUVECs as well as by strong positive EPOR protein staining in vascular endothelium in vivo [10,11,12,13]. Moreover, EPO stabilizes vascular integrity, increases the number of ECs, protects these cells against ischemia and apoptosis [14,15,16,17,18], and stimulates angiogenesis in vitro and in vivo [9,11,19,20,21,22]. The presence of EPOR in ECs was also shown by Yamaji et al. [23], who suggested that besides full-length EPOR, brain capillary ECs also express soluble EPOR (sEPOR), and that EPO acts directly on brain capillary ECs as a competence factor.

In this article we review the effect of EPO on ECs in ischemic heart neovascularization and in retinal revascularization of injured vasculature, as well as its effect on neural progenitor cells, on tumor angiogenesis, and in other pathological conditions.

### 1.1. Endothelial Cells

The EPO-induced proangiogenic phenotype of human ECs, EA.hy926, was demonstrated by Ribatti et al. [22]. EA.hy926 cells presenting EPOR directly interact with EPO, which is followed by phosphorylation of JAK-2 and STAT-5 [9], cell proliferation, production of matrix metalloproteinase-2 (MMP-2), and vascular differentiation. Indeed, the angiogenic response of ECs in chick embryo chorioallantoic membrane in vivo is quantitatively and qualitatively similar to that developed by the prototypic angiogenic fibroblast growth factor 2 (FGF-2) [22], while a previous observation declared that recombinant EPO (rhEPO)-induced blood vessel growth in vitro (rat aorta ring assay) is partially dependent on endothelin-1 (ET-1) [11]. However, in contrast to this finding, Ribatti et al. [22] and Hu et al. [24] proved that ET-1-produced ECs are unable to stimulate the growth of new blood vessels.

The study of Ashley et al. [25] confirmed that rhEPO stimulates proliferation and/or vasculogenesis of microvascular endothelial cells (MVEC) from neonatal rat mesentery on the hormone-rich Matrigel substrate as well as on the extracellular matrix protein type I collagen. Their study was the first showing the effect of EPO on the endothelium of the neonatal gastrointestinal tract and supposing the role of EPO as an endogenous stimulant of the vessel growth in neonatal gastrointestinal development. In addition, in the presence of EPO, Matrigel tubules were qualitatively more complex, stable, and more frequently formed compared to Matrigel tubules in the presence of vascular endothelial growth factor (VEGF) and FGF-2 [25]. Furthermore, ECs from bovine aorta were stimulated by rhEPO in a concentration-dependent manner and revealed strong EPO-induced upregulation of *kinase domain receptor* (*KDR*, encoding *VEGF receptor 2*, *VEGFR-2*) and *fms related tyrosine kinase 1* (*flt-1*, encoding *VEGFR-1*) gene expression. On the other hand, a remarkable inhibition of EPO proliferative effect by anti-VEGF antibody and the finding that the addition of VEGF to the medium in the absence of fetal calf sera is sufficient to induce proliferative activity of EPO, highlighted the crucial importance of VEGF in the effect of EPO on ECs. The relationship between EPO and VEGF can be particularly important in the normal process of neovascularization and in patients treated with rhEPO [26].

**Figure 1:**
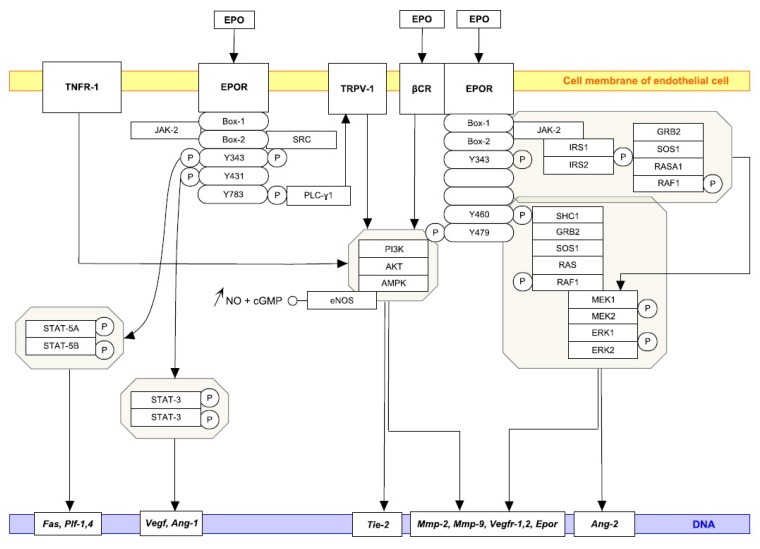
EPO and the signalization of ECs. EPO-induced signalization of EC along with target genes associated with angiogenesis are outlined. Docking sites for several signaling proteins are marked with P; only positive interactions are presented with full black arrows. EPOR WikiPathway (Available on: http://www.wikipathways.org) was modified with PathVisio tool based on the references mentioned in the article.

Besides a direct stimulatory effect of EPO on different ECs (human bone marrow endothelial cells, HUVECs, and human umbilical artery endothelial cells), EPO also induces EPOR expression at low oxygen pressure [27]. Furthermore, in response to EPO the expression of nitric oxide (NO) synthase [28] is also elevated and is followed by subsequent NO and cGMP production. Beleslin-Cokic et al. [27] provided evidence that hypoxia increases the capacity of ECs to produce NO. If we go deeper into the molecular mechanism, EPO induces Ca^2+^ influx in bovine aortic ECs via activation of phospholipase C-γ1 signaling pathway, which leads to the activation of transient receptor potential vanilloid type 1 (TRPV1), followed by the activation of the serine/threonine kinase AKT and AMP-activated protein kinase (AMPK) and the phosphorylation of eNOS. A TRPV1–AKT–AMPK–eNOS complex then leads to an increase in NO bioavailability and ultimately to angiogenesis [29]. Similarly, EPO-sustained release of gelatin hydrogen microspheres improved blood perfusion of ischemia limb in mice via increasing capillary and arteriolar densities mediated by upregulation of EPOR and the activation of AKT–eNOS–MMP-2 signaling pathway [30]. Interestingly, in ECs the β common receptor (βCR) can also play an integrative role in EPO-mediated activation of eNOS. In this regard, AMPK mediated the EPO-induced increase in the phosphorylation of βCR and the production of a βCR–AMPK–eNOS complex, which is followed by increased NO production and angiogenesis [31] (Figure 1).

### 1.2. Bone Marrow

EPOR is expressed in ECs derived from bone marrow of patients with monoclonal gammopathy of undetermined significance (MGUS) and patients with multiple myeloma (MM) (MGECs and MMECs, respectively). Moreover, EPOR is over-expressed also in bone marrow-derived macrophages (BMMAs) from MM compared to MGUS patients. Interestingly, the conditioned media of MGECs, MMECs as well as BMMAs induce a strong angiogenic response in vivo in the chorioallantoic membrane assay. Indeed, the data of Lamanuzzi et al. [32] and de Luisi et al. [33] signify the effect of EPO on MGECs and MMECs and BMMAs, respectively, and confirm the role of EPO/EPOR pathway in the regulation of the angiogenic response of MM patients. Although expression of hypoxia-inducible factor 1α (HIF-1α) and EPOR does not differ between chronic mountain sickness patients and control subjects, the expression of HIF-2α and EPO is higher in the bone marrow cells of chronic mountain sickness patients than in controls. Higher microvascular density, which is involved in the pathogenesis of this disease, could be stimulated by HIF-2α/EPO pathway of the bone marrow cells (autocrine and/or paracrine mechanisms) in the bone marrow niche [34]. Furthermore, EPO stimulates biosynthesis of proteoglycan together with the upregulation of chondrogenic marker genes including *Sry-related HMG box* (*Sox*) *Sox-5, Sox-6, Sox-9*, *collagen type 2* and *aggrecan* [35]. Indeed, the results of Wan et al. [35] and Holstein et al. [36] showed that EPO enhances chondrogenic and angiogenic responses during bone repair and could serve as a therapeutic agent to support skeletal regeneration.

### 1.3. Adipose Tissue

Expression of EPOR in adipocytes is important for pharmacological EPO activation of a variety of signaling pathways, which are not responsive to physiological levels of EPO [37]. Indeed, in 3T3-L1 adipocytes containing EPOR, EPO stimulates the glucose uptake and potentiates the effect of insulin via an AKT-dependent mechanism in vitro and in vivo [38]. Moreover, EPO treatment increases the expression of angiogenic factors, enhances microvascular density, and reduces inflammation and apoptosis of human fat tissue that has been injected into nude mice [39], and reverses microvascular dysfunction during wound healing in hypercholesterolemic mice [40]. On the other hand, although mice with EPOR expression restricted to hematopoietic tissue become obese and insulin-resistant, and have decreased levels of proopiomelanocortin in the hypothalamus [41], adipose tissue-specific disruption of EPOR does not change (under the physiological levels of EPO) adipose expansion, adipocyte morphology, insulin resistance, inflammation, or angiogenesis in vivo [37].

### 1.4. Heart

Westenbrink et al. [42] demonstrated that EPO could stimulate neovascularization and improve cardiac function in ischemic hearts. The mechanism is probably controlled by local *Vegf* and *Epor* expression Indeed, EPO regulates normal endothelial progenitor cells (EPCs)-mediated turnover and improves microvascularization and cardiac function. Moreover, so-called “homing” (migration of circulating stem or progenitor cells into target tissue) of EPCs into the ischemic myocardium was observed, where ischemia is essential for the switching from a vascular “state” to EPO-induced neovascularization [42]. Other studies demonstrated upregulation of VEGF, which is sustained at sites of EPO-induced neovascularization [43,44]. On the contrary, mice lacking *Epor* expression (−/−) show reduced neovascularization (without upregulation of VEGF) with decreased homing of EPCs and bone marrow-derived proangiogenic cells in ischemic hind limbs [45]. In this regard, VEGF as an angiogenic factor is able to stimulate the proliferation of ECs in situ and also has chemotactic effects on EPCs [46]. Moreover, the presence of ischemia and hypoxia together significantly increases the sensitivity of ECs to EPO by exponential increase of *Epor* expression [27]. Indeed, the vascular EPO/EPOR system plays a crucial protective role against hypoxia/ischemia, providing a new therapeutic target in cardiovascular medicine [47]. The physiological effects of EPO and the mechanism of EPO-induced cardioprotection together with a therapeutic perspective on EPO in patients with myocardial infarction are very aptly described in the paper of Sanchis-Gomar et al [48].

Furthermore, Nakano et al. [45] demonstrated that EPOR may play an important role in VEGF secretion, mobilization of EPCs, and angiogenesis of ischemic tissue, as well as in the presence of EPO in peripheral vasculature. Their study showed an important role of EPO in the upregulation of VEGF/VEGFR-2 system for mobilization of EPCs in local ischemic tissue. They really indicated that vascular EPOR as well as EPO promotes post-ischemic angiogenesis via increase of VEGF secretion from ischemic muscle, EPCs mobilization, and recruitment of bone marrow-derived proangiogenic cells to the ischemic tissue [45]. In the infarcted area of the heart, EPO-gelatin hydrogel drug delivery system (EPO-DDS) increases the level of EPO, but has little or no effect on the serum level of EPO. This means that the effect of EPO-DDS is solely local and enhances angiogenesis through neovascularization [49]. Interestingly, pre-incubation of bone marrow mesenchymal stem cells in hypoxic conditions before transplantation into an infarcted heart increases the expression of pro-angiogenic and pro-survival factors including HIF-1α, angiopoietin-1 (Ang-1), VEGF, VEGFR-2, EPO, B-cell lymphoma 2 (Bcl-2), and B-cell lymphoma-extra large (Bcl-xL), followed by upregulation of angiogenesis. Indeed, stem cell therapy with hypoxic pre-conditioning is more beneficial than normoxic, from both a morphologic and a functional point of view [50]. Kobayashi et al. [49] confirmed that EPO-DDS induced an increase of both CD31-positive microvessels as well as myocardial VEGF expression, and emphasized the critical role of VEGF (angiogenesis promotor) and Bcl-2 (apoptosis inhibitor) upregulation in the early phase of post-myocardial infarct.

EPO is also able to prevent the pathological changes observed during diabetic cardiomyopathy. In this regard, EPO treatment increases both the number of peripheral blood EPCs and the expression of EPOR and VEGF in left ventricular myocardial tissue, followed by improved revascularization and the inhibition of cardiac fibrosis in diabetic rats [51].

### 1.5. Leg Ischemia

The problem of revascularization is critical in leg ischemia, and conventional methods of revascularization often fail [52]. EPO [21] and EPO-primed endothelial colony-forming cells [53] might play a significant role in leg ischemia therapy through increasing blood flow and angiogenesis. Furthermore, recently synthesized nonhematopoietic derivatives of EPO allow both a higher tissue protective effect as well as elimination of an unwanted increase of hematocrit [52]. Interestingly, angiogenesis of ischemic limbs induced by the abovementioned EPO-DDS is mediated through the AKT/eNOS pathway and MMP-2 activation [54], which was associated with the cell proliferation of ECs and the subsequent proliferation of vascular smooth muscle cells [55,56]. Moreover, increased platelet activity and their adhesion appear to be very important for the EPO mediated process of neovascularization from hind-limb ischemia and for the release of VEGF and stromal cell-derived factor (SDF-1) [57]. It has been demonstrated that P-selectin antibody blocked EPO-mediated angiogenesis and platelet adhesion to the ECs followed by decrease in plasma VEGF and SDF-1 levels [57].

### 1.6. Retinopathy

Chen et al. [58] described how EPO deficiency can support the development of retinopathy; however, early EPO supplementation can compensate for its deficiency and promote neural and vascular survival. On the other hand, late EPO treatment does not have any protective effect on retinal vasculature during the neovascularization phase of retinopathy, but can worsen pathological proliferation. EPO stimulates the transport of EPCs from bone marrow into the retina, their differentiation into ECs, and the revascularization of injured vasculature of the retina [59,60]. It also increases the number of proangiogenic microglia or macrophages in the retina and plays a critical role in retinal vascular growth and repair [61,62]. Multivariate regression analysis indicates that EPO and VEGF are independently associated with proliferative diabetic retinopathy. Surprisingly, EPO shows a stronger association with diabetic retinopathy than VEGF. In this regard, sEPOR inhibits EPO-induced retinal neovascularization in vitro and in vivo [63]. Similarly, intravitreous injection of EPO siRNA inhibits endogenous expression of EPO in the retina, followed by the suppression of destructive vessel proliferation during the neovascular phase of retinopathy [64]. Since the level of EPO is increased during the development of retinopathy, inhibition of *Epo* expression by siRNA might serve as a potential therapeutic intervention in proliferative retinopathy [64]. Based on the paper of Eldweik and Mantagos [65], the role of the VEGF inhibitor in the treatment of retinopathy of prematurity is also evident. Wijngaarden et al. [66] demonstrated very interesting strain-related differences in the expression of *Vegf*, *Epo*, *Vegfr-2*, *Ang-2*, *Insulin-like growth factor 1* (*Igf-1*), and other genes that are significantly upregulated in the retina of the sensitive to oxygen-induced retinopathy strains (SPD) and DA rat neonates compared to resistant F344 strain after exposure to cyclic hyperoxia. Thus, expression of genes involved in angiogenesis is significantly higher in the SPD and DA strains compared to the resistant one F344. Indeed, the sensitivity to oxygen-induced retinopathy correlates better with differential gene expression during periods of cyclic hyperoxic exposure than during subsequent persistent hypoxia. Such fluctuations between hyperoxia and hypoxia are considered the main mediators of retinopathy of prematurity in rats and might have potential relevance for humans [66].

Interestingly, data from Caprara et al. [67] suggested that EPOR is not necessary for the maturation, function, and survival of rod photoreceptors, Müller cells, and amacrine, horizontal and ganglion cells of the peripheral retina. Indeed, retinal angiogenesis and vasculature are normal in the absence of EPOR (flox/flox; α-Cre mice). On the other hand, using a rat model of oxygen-induced retinopathy, Yang et al. [68] demonstrated that VEGF-A activates EPOR and enhances VEGFR-2-mediated pathological angiogenesis in the retina. Activated EPOR was found in ECs of the retina at postnatal day 18, when pathological angiogenesis in the form of intravitreal neovascularization occurred.

Although rhEPO together with recombinant IGF were tested for the ability to prevent the loss of vasculature during the first phase of retinopathy of prematurity, studies on the role of genetic components, NO, adenosine, apelin, and β-adrenergic receptor have opened up new opportunities for retinopathy treatment [69].

### 1.7. Brain

Wang et al. [44] disclosed that rhEPO induces the secretion of VEGF in neural progenitor cells via phosphoinositide 3-kinase PI3K/AKT and mitogen-activated protein kinase ERK1/2 signaling followed by the upregulation of *Vegfr-2* expression in cerebral ECs and the promotion of angiogenesis. The same group demonstrated that rhEPO starting 24 h after experimental stroke in the rat significantly improved functional recovery and enhanced angiogenesis and neurogenesis, which correlated well with increased levels of brain-derived neurotrophic factor (BDNF) and VEGF. It seems that EPO provides a permissive microenvironment for neuronal plasticity during stroke recovery. In this regard, Li et al. [70] confirmed that intraperitoneal administration of rhEPO after focal ischemia reduces cell death of ECs, enhances angiogenesis, and clearly restores the local cerebral blood flow. In fact, neovascular protection and angiogenesis are supported on a molecular level by increased expression of angiopoietin *receptor Tie-2*, *Ang-2*, *Vegf*, and *Epor* in vascular ECs of the penumbra region. Furthermore, Li et al. [71] also demonstrated significant rhEPO-induced protection against blood–brain barrier leakage, reduction of blood–brain barrier permeability, and reduction of brain edema, which are usually induced by focal ischemia in the acute phase after injury. In contrast to upregulated VEGF protein, its receptors VEGFR-1 and VEGFR-2 significantly decreased after rhEPO on the third day after injury. EPO-mediated prevention of blood–brain barrier disruption after ischemia might involve the storage of ECs, preservation of microvasculature integrity, and downregulation of VEGFR-1 and VEGFR-2 receptors.

Increased VEGF expression and activated VEGF/VEGFR-2 signaling pathway after rhEPO administration also resulted in an improvement of brain repair after anoxia. Indeed, rhEPO enhances angiogenesis, reduces white matter damage, and promotes cognitive recovery in anoxia rats [72]. Furthermore, EPO mediates neurovascular remodeling and neurobehavioral recovery in rats after traumatic brain injury via increased brain VEGF expression and phosphorylation of VEGFR-2 [73]. In addition, Wang et al. [74] provided proof of crosslink evidence between tumor necrosis factor α (TNF-α) and EPOR in cerebral ECs in vitro. Suggested interaction of TNF-α with TNF-α receptor 1 (TNFR-1) sensitizes cerebral ECs for EPO-induced angiogenesis by upregulation of EPOR, which boosts the effect of EPO on the activation of VEGF/VEGFR-2 and Ang-1/Tie-2 signalization.

EPO-deficient mice (EPO-TAg^h^) indicate an increase in both mRNA as well as protein levels of HIF-1α, VEGF, EPOR, pSTAT-5/STAT-5 ratio, and eNOS, together with higher cerebral capillary density under normoxic conditions. Similarly, wild-type [75] mice show the same increase in the expression of hypoxia-related genes as well as increased capillary density after acute hypoxia, without any additional changes under chronic hypoxia. On the other hand, chronic hypoxia except for NO metabolites reduces the expression of HIF-1α, VEGF, EPOR, and pSTAT-5/STAT-5 ratio in EPO-TAg^h^ mice. Although in EPO-TAg^h^ mice cerebral angiogenesis develops through the HIF-1α/VEGF pathway under normoxic conditions, the neuroprotective and angiogenesis pathways in EPO-TAg^h^ mice seem to be altered [76]. Recently, Pichon et al. [77] demonstrated via EPO-TAg^h^ model that EPO can play a key regulating role in the neural control of ventilatory acclimatization to hypoxia and hypoxic ventilatory response. Moreover, chronic EPO deficiency of EPO-TAg^h^ mice induces cerebral and cardiac angiogenesis, which might have synergic effects not only in neuro- and cardioprotection but also in optimization of the O_2_ supply.

Interestingly, it was exposed that EPO augments the angiogenesis and reduces the expression of the receptor for advanced glycation end products (RAGE) in the brains of aged Tg2576 mice. This evidence pointed out the effect of EPO on improved memory and mitigating endothelial degeneration induced by amyloid-β in an Alzheimer’s disease model [78].

Furthermore, based on the promising scientific results, preclinical data and phase II and III clinical trials are currently in process to determine the safety and efficacy of neuroprotective dosages of EPO at extreme prematurity and hypoxic–ischemic encephalopathy in neonates [79].

### 1.8. Tumors

The study of Yasuda et al. [80] revealed that the normal human cervix and malignant endometrial and ovarian tumors produce EPO and EPOR, and that the tumor cells and capillary ECs themselves are sites responsive to the EPO. Yasuda et al. [81] proposed the presence of a paracrine or autocrine EPO–EPOR loop and its contribution to tumorigenesis in female reproductive organs, which was based on the fact of the mitogenic action of EPO as well as on the finding that injection of sEPOR or EPO-monoclonal antibody into blocks of tumor specimens is followed by apoptosis of tumor and ECs. Furthermore, local rhEPO application in window chambers or stable expression of a constitutively active EPOR mutant (EPOR-R129C) might have a potential to stimulate tumor angiogenesis, associated with significant stimulation of tumor growth [82]. On the other hand, co-injection of a neutralizing anti-EPO antibody and rhEPO or targeting endogenous EPO using sEPOR inhibits the initiation of tumor angiogenesis of rat R3230-GFP mammary tumor cells and leads to growth delays during the initial stages of tumorigenesis. The listed results clearly demonstrate the role of EPO in both growth and angiogenesis of tumors, and as a key protein contributing substantially to the development of almost all malignancies [83]. In this regard, EPO-mimetic peptide 9 (EMP-9) decreases the angiogenesis of the stomach, choriocarcinoma SCH, and melanoma P39, but does not affect the vessels in the host. It seems that EMP-9 damages only proliferating ECs of capillaries, but not static vessels. On the other hand, EMP-1 stimulates angiogenesis through the phosphorylation of STAT-5 protein [83].

Although some studies have not confirmed a direct stimulatory effect of EPO on tumor cells, there is ample evidence of this effect on the proliferation of ECs and/or angiogenesis of tumors. In this regard, EPO induces angiogenesis in chemically induced murine hepatic tumors [84] and accelerates the growth of EPOR-negative Lewis lung carcinoma cells by promoting tumor angiogenesis in vivo [85].

Indeed, an EPO analogue stimulates neovascularization in colorectal liver metastases of hepatectomized and non-hepatectomized mice [86]. Moreover, Nico et al. [87] demonstrated that EPO secreted by glioma tumor cells affects glioma vascular ECs and promotes angiogenesis in a paracrine manner. The specificity of EPO was confirmed via an anti-EPO antibody, which significantly inhibited the angiogenesis response. Despite the absence of melanoma growth stimulation in vivo, EPO increased vascular size in the xenografts. EPO induced angiogenesis in Matrigel plug assays, whereas neutralization of EPO secreted by melanoma cells resulted in decreased angiogenesis, which supports the role of EPO/EPOR in melanoma progression via stimulation of angiogenesis [19]. Even more interestingly, EPO accelerated the tumor growth of rat prolactinoma MMQ pituitary adenoma xenografts lacking EPOR via the enhancement of angiogenesis in vivo, without a direct effect of EPO on MMQ cells in vitro. The application of EPO increased the phosphorylation of JAK-2 and STAT-3 and the expression of VEGF in HUVEC cells in vitro and in MMQ pituitary cell xenografts in vivo. Yang et al. [19] found that EPO administration promoted the growth of pituitary adenomas by enhancing angiogenesis via the EPO–JAK-2–STAT-3–VEGF (Figure 1) signaling pathway and should be used with caution in anemia patients bearing pituitary adenoma due to its potentially deleterious effects. On the contrary, preoperative administration of EPO stimulated tumor recurrence in an animal model of colon cancer without evidence of increased angiogenesis or enhanced cell proliferation [88]. Importantly, EPO/EPOR levels correlated well with angiogenesis and progression of patients with hepatocellular, squamous cell of the tongue and non-small cell lung carcinomas, neuroblastoma, melanoma, and gastric adenocarcinoma [89,90,91,92,93,94,95]. On the other hand, it was found that EPO-independent EPOR-signaling pathway plays a potential role in the cell proliferation and angiogenesis of human pterygium [96].

In addition, Ribatti et al. [89,97] showed a correlation between the stage of carcinoma and microvascular density. They observed a higher degree of vascularization in stage IV of gastric carcinoma as well as in badly differentiated hepatocellular carcinoma. Recombinant EPO induced a proangiogenic phenotype that involves early (i.e., increase of cell proliferation and MMP-2 production) and late (differentiation into vascular tubes) angiogenic events in human ECs. In patients with gastric and hepatocellular carcinomas, the levels of EPO/EPOR correlate with angiogenesis and tumor progression; therefore, EPO might serve as an endogenous stimulant of vessel growth by an autocrine and/or paracrine loop.

Very recently, Tankiewicz-Kwedlo et al. [98] demonstrated an EPO-stimulating effect on the proliferation of EPOR-positive human colorectal adenocarcinoma cells DLD-1 in vitro as well as on the angiogenesis of DLD-1 xenografts in vivo. Moreover, EPO increased VEGFR-1 expression, which correlates well with EPOR and VEGF expression in DLD-1 xenografts. Similarly, EPOR and microvessel density significantly correlated with the expression of adrenomedullin, the new metastasis-related and angiogenic regulatory factor involved in tumor angiogenesis, and the recurrence and metastasis of hepatocellular carcinoma and other tumors [99]. On the other hand, it was pointed out that EPO in co-therapy with carboplatin enhances vascularization and perfusion, improves the delivery of carboplatin in non-small cell lung cancer xenografts, and results in more pronounced apoptosis. Similar observations of reduced tumor growth due to increased drug accumulation were obtained in squamous cell and colon carcinoma xenografts co-treated with 5-Fluoruracil and EPO [100].

The lymph node as a new target of EPO was presented by Lee et al. [101]. They showed that EPO can stimulate both lymph node lymphangiogenesis and nodal metastasis by increased migration, capillary-like tube formation, and dose- and time-dependent proliferation of human lymphatic ECs in tumor-bearing animals. Intraperitoneal administration of EPO induced AKT and ERK1/2 signalization followed by peritoneal lymphangiogenesis stimulation. Furthermore, systemic treatment of EPO increased infiltration of CD11b^+^ macrophages in tumor-draining lymph nodes and also increased VEGF-C expression in lymph-node-derived CD11b^+^ macrophages as well as in bone-marrow-derived macrophages in a dose- and time-dependent manner.

## 2. Conclusions

Various in vitro and in vivo studies have demonstrated the potential of EPO to stimulate ECs growth in various tissues and tumors. EPOR expression in ECs together with JAK-2 and STAT-5 signal activation confirmed the EPO/EPOR-mediated pro-angiogenesis potential. Furthermore, EPO has the ability to induce neovascularization via expression and upregulation of VEGF/VEGF-R, essential growth factors for vascular ECs and potent angiogenic factors.

Based on preclinical and clinical trials, rhEPO treatment is a promising future agent for ischemic heart protection, leg ischemia therapy, bone repair, retinopathy treatment, and brain neuroprotection.
